# Visceral adiposity-related dietary patterns and the risk of cardiovascular disease in Iranian adults: A population-based cohort study

**DOI:** 10.3389/fnut.2022.812701

**Published:** 2022-07-28

**Authors:** Nazanin Moslehi, Fatemeh Rahimi Sakak, Maryam Mahdavi, Parvin Mirmiran, Fereidoun Azizi

**Affiliations:** ^1^Nutrition and Endocrine Research Center, Research Institute for Endocrine Sciences, Shahid Beheshti University of Medical Sciences, Tehran, Iran; ^2^Obesity Research Center, Research Institute for Endocrine Sciences, Shahid Beheshti University of Medical Sciences, Tehran, Iran; ^3^Department of Clinical Nutrition and Dietetics, Faculty of Nutrition and Food Technology, National Nutrition and Food Technology Research Institute, Shahid Beheshti University of Medical Sciences, Tehran, Iran; ^4^Endocrine Research Center, Research Institute for Endocrine Sciences, Shahid Beheshti University of Medical Sciences, Tehran, Iran

**Keywords:** reduced rank regression, partial least squares regression, dietary pattern, cardiovascular disease, hybrid method

## Abstract

**Background:**

Visceral obesity is a significant predictor of cardiovascular disease (CVD). Diet may associate with CVD risk through its effects on visceral adiposity. This study aimed to find dietary patterns (DPs) related to indicators of visceral adiposity and to determine whether the DPs were associated with CVD risk.

**Methods:**

This prospective study included 2,496 participants of the Tehran Lipid and Glucose Study (TLGS) without CVD, who were followed from the third study examination (2005–2008; baseline) to March 2018. DPs at baseline were determined using reduced rank regression (RRR) and partial least squares regression (PLS). The response variables were age and BMI-adjusted waist circumference (WC) and age-adjusted visceral adiposity index (VAI).

**Results:**

Two and three DPs were retained with RRR and PLS, respectively. The first patterns of each method were mainly characterized by adjusted-WC (RRR: 10.8%, PLS: 8.6%); none of them were associated with CVD risk. The second pattern of RRR and the third pattern of PLS were mainly explained by adjusted-VAI (RRR: 3.3, PLS: 2.1%). After adjusting for CVD risk factors, the hazard ratios [95% confidence intervals (CI)] for CVD in the second and third tertiles of the RRR-pattern 2 were 1.76 (1.15, 2.69) and 1.55 (1.00, 2.43) vs. the first tertile (*p*-trend: 0.058). This pattern had high positive loadings for non-leafy vegetables, pickled vegetables, fried vegetables, and bread and high negative loadings for eggs, cakes, butter, jam-honey, red meat, poultry, fish, juice, non-fermented dairy, and fruits. Per one SD increase in PLS-pattern 3 score, the risk of CVD was 19% higher (95%CI = 3–38%). This positive association was also observed across tertiles of the pattern (p-trend: 0.032). This pattern was characterized by high intakes of leafy vegetables, non-leafy vegetables, organ meat, soft drinks, olive oil, pickled vegetables, fried vegetables, and bread and low intakes of biscuits, cakes, butter, eggs, and non-fermented dairy.

**Conclusion:**

For each of the RRR and PLS approaches, a visceral-related DP that was positively linked to CVD was identified. These two patterns had a modest correlation. The pattern generated by PLS explained more variations in food groups and offered stronger evidence of association with CVD than the RRR-derived pattern.

## Introduction

Cardiovascular disease (CVD) is the most common cause of mortality worldwide. According to World Health Organization estimates, 17.9 million individuals died from CVD in 2019 (1). Dietary risk factors showed the highest contributions to CVD deaths and CVD disability-adjusted life years among other non-communicable diseases worldwide. In 2017, 10 million CVD-related deaths and 207 million CVD disability-adjusted life years globally were attributed to an unsatisfactory diet (2). The majority of the dietary risk factors for CVD were proposed based on the findings of researches examining the associations between individual foods and nutrients (3, 4). Recent scientific statements intended to improve cardiovascular health, on the contrary, highlight the significance of dietary patterns (DPs) beyond individual foods or nutrients. According to the statements, cardio-protective DPs are characterized by high intakes of fruits, vegetables, whole grains, and healthy sources of protein and low intakes of processed foods and added sugar and salt beverages and foods (5, 6). However, the majority of the information comes from studies conducted on Western populations; hence, non-Western studies would aid in the development of future dietary recommendations (6).

Dietary pattern analysis is a supplementary approach that allows researchers to look at the whole diet rather than just a single item or nutrient. The approach captures the cumulative impact of dietary components by accounting for food-nutrient interactions (7). Dietary pattern analysis is classified into three types, namely, hypothesis-driven approaches, exploratory approaches, and hybrid approaches combining the two (8, 9). Reduced rank regression (RRR) and partial least squares (PLS) are two-hybrid methods in which DPs are derived based on intermediate response variables. The response variables are biomarkers of exposure or disease or other mediating variables thought to be essential for the development of the disease. The methods enable food pattern detection to include *a priori* assumptions about possible pathophysiological pathways (8, 9). To date, limited prospective studies investigate the association between RRR-derived DPs and risk of CVD morbidity and mortality (10–17), and only two studies employed the PLS approach in addition to the RRR method (13, 17). In these studies, dietary intakes of nutrients (e.g., nutrients related to excess energy intake), body mass index (BMI), blood pressure, lipid profiles, and inflammatory markers were selected as response variables (10–17). Visceral adiposity may be an important mediator in the relationship between diet and CVD risk (18, 19). However, the association between visceral adiposity-related DPs and CVD risk has yet to be investigated. To address this, we determined DPs related to waist circumference (WC) and visceral adiposity index (VAI), the two visceral adiposity indices. We then examined the association between the adiposity-related patterns and incident CVD over a mean of 10.2 years of follow-up.

## Materials and methods

### Study population

**FIGURE 1:**
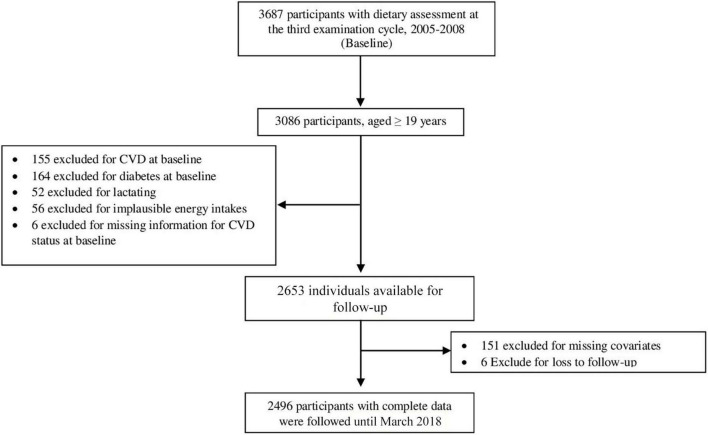
Diagram showing the selection process of the study participants.

This longitudinal study was conducted within the Tehran Lipid and Glucose Study (TLGS) framework, a large-scale population-based prospective study being performed on a representative sample of Tehran, the capital of Iran. Participants were recruited from residents of District 13 of Tehran. Details, rationale, sampling, and data collection methods of TLGS have been mentioned previously (20). In brief, the TLGS began in 1999–2001; the data gathering process is ongoing at 3-year intervals to update the health-related baseline measurements of participants. Since the third examination cycle (2005–2008), dietary information has been gathered using a food frequency questionnaire (FFQ). Of the 3,687 participants in the third examination cycle who had complete dietary data, which we used as the baseline for the current analyses, those aged ≥ 19 years (*n* = 3,086) were selected. Participants with prevalent CVD or diabetes, lactating women, those who had reported implausible energy intakes [<814 (first percentile) or > 5,320 (99th percentile) for women; <915 (first percentile) or > 6,641 kcal (99th percentile) for men], and those with missing data about CVD status at baseline or follow-ups or missing covariates were excluded. A total of 2,496 adults free of CVD at the third examination cycle (as baseline) were finally included in this study, who were followed until March 2018 (Figure 1).

This study was conducted based on the guidelines of the Declaration of Helsinki; the Research Institute for Endocrine Sciences, Shahid Beheshti University of Medical Sciences, approved the study design. All participants provided written informed consent before participation in the study.

### Demographic and anthropometric measurements

Information on age, sex, education, smoking habits, past medical history of CVD, drug usage, and family history of premature CVD of participants was collected by trained investigators using a pretested questionnaire in private and face-to-face interviews. The participants were weighed using a digital scale while wearing less clothing and no shoes, and their height was measured in a standing position with no shoes using a non-stretched tape measure. BMI was calculated by dividing weight in kilograms by height squares in meters. WC was measured by using a non-stretched tape with no pressure on the body surface. Leisure time, job, and household activities in the past year were assessed using the Persian-translated Modifiable Activities Questionnaire (21), and total physical activity was reported as metabolic equivalent minutes per week (MET-min/wk).

### Clinical and biochemical measurements

Systolic and diastolic blood pressure was measured two times in a seated position on the right arm after 15 min of rest, using a standardized mercury sphygmomanometer (Riester, Jungingen, Germany). The mean of the two measurements was reported as the participants’ blood pressure.

Fasting blood samples were collected in the morning after 12–14-h overnight fasting based on the standard protocol. All biochemical measurements were assessed at the TLGS research laboratory on the same day of blood collection. Fasting plasma glucose (FPG), triglycerides (TGs), total cholesterol, and high-density lipoprotein cholesterol (HDL-C) were measured using laboratory kits (Pars Azmun, Tehran, Iran) (22).

### Dietary assessment

Dietary data at baseline were collected using a validated and reliable semi-quantitative FFQ (23, 24). The questionnaire had 168 food items with predefined portion sizes. A trained dietitian asked participants to report the consumption of each food item according to its predefined portion size during the preceding year on a daily, weekly, or monthly basis as appropriate. The portion size of consumed foods was converted to daily frequency and then converted to grams. On the basis of their nutrient profiles, the ingested items were classified into 34 food groups for DP analysis (Supplementary Table 1).

### Intermediate (response) variables for dietary pattern extraction

The intermediate variables to extract DPs were WC and VAI. The VAI is a sex-specific index that incorporates anthropometric (BMI and WC) and biochemical variables (TG and HDL) to indirectly assess visceral adiposity (25). It can be calculated using the following formulas (25):


$$\begin{array}{c} \mathrm{Male}:\left[\mathrm{WC}/\left(39.68+\left(1.88\times \mathrm{BMI}\right)\right)\right]\times \left(\mathrm{TG}/1.03\right)\\ \times \left(1.31/\mathrm{HDL}\right) \end{array}$$

$$\begin{array}{c} \mathrm{Female}:\left[\mathrm{WC}/\left(36.58+\left(1.89\times \mathrm{BMI}\right)\right)\right]\times \left(\mathrm{TG}/0.81\right)\\ \times \left(1.52/\mathrm{HDL}\right) \end{array}$$

To control the effects of age and BMI on the visceral adiposity variables, WC was adjusted for age and BMI, and VAI was adjusted for age by the residual method.

### Definition of outcome and terms

The data collection process for CVD outcomes was previously addressed (26). Participants were followed annually to identify medical events. After reporting a medical event by participants or their relatives, an expert panel evaluated the medical documents to confirm the incidence of a specific outcome. The CVD incidence was defined as myocardial infarction (definite or probable), angiographically proven coronary heart disease, stroke, and death due to cardiovascular causes. Any CVD events in first-grade female family members aged 65 years or lower or first-grade male family members aged 55 years or lower were considered a premature family history of CVD (27). Based on the American Diabetes Association’s guidelines, diabetes mellitus was defined as FPG ≥ 126 mg/dl or 2-hPG ≥ 200 mg/dl or glucose-lowering medical drug usage (28). Hypertension was defined as having a systolic blood pressure ≥ 140 mmHg or a diastolic blood pressure ≥ 90 mmHg or being on antihypertensive medication (29). Hypercholesterolemia was defined as serum total cholesterol ≥ 200 mg/dl or the use of any lipid-lowering medical drug. We categorized participants according to their smoking habits into two groups, namely, ever-smokers (current smokers or ex-smokers) and non-smokers. We divided education status into ≤ 12 years and > 12 years (academic education).

### Statistical methods

Energy-adjusted food groups, age and BMI-adjusted WC, and age-adjusted VAI were determined using the residual method. Since the distribution of food groups and VAI were skewed, the natural logarithmic transformation was performed on the variables before residual adjusting. DPs were obtained by two statistical methods, namely, RRR and PLS, using PROC PLS in SAS version 9.1.3. Energy-adjusted intakes of the 34 groups were selected as predictors, and adjusted-WC and adjusted-VAI were selected as intermediate variables. For further statistical analyses, the two patterns of RRR and the first three patterns of PLS that explained the most variations in intermediate variables were retained.

Participants were divided into three groups based on the tertiles of dietary pattern score derived by the first pattern obtained for each method. The characteristics of participants across the tertiles of the pattern scores were compared using ANOVA for normally distributed continuous variables, the Kruskal-Wallis test for non-normally distributed continuous variables, and chi-square for categorical variables.

We used the Cox proportional hazards regression model to estimate CVD risk per one SD increase in each dietary pattern score. CVD risk was also assessed for the tertile of each pattern, considering the first group as the reference category. A linear trend (*p*-trend) was obtained by assigning the median value to each tertile and treating this as a continuous variable. Statistical tests and graphical diagnostics based on scaled Schoenfeld residuals were used to assess the proportional hazards (PH) assumption. All proportionality assumptions were generally satisfied. The time of follow-up was calculated from the date of entrance to the study to the first occurrence of CVD events or the last follow-up date. Regression models were adjusted for potential covariates related to the risk of CVD. In the first adjusted model, age (continuous), sex, premature family history of CVD (yes/no), and energy intake (continuous) were included as covariates. The second model was additionally adjusted for academic education (yes/no), ever-smoking (yes/no), physical activity (continuous), prevalent hypertension (yes/no/missing), prevalent hypercholesterolemia (yes/no), and baseline fasting plasma glucose (continuous). Six individuals lacked the information necessary to assess their hypertension status at baseline, and thus, we created an additional category of “missing” for them. Results were reported as hazard ratios (HR) and 95% confidence intervals (95%CI).

In sensitivity analyses, the risk of CVD was recalculated after excluding the following: 1, those with early CVD events (< 2 years); 2, excluding participants aged < 30 years; 3, those with early CVD events and aged < 30 years. Statistical analyses were performed using SPSS version 20; a *p*-value < 0.05 was considered statistically significant.

## Results

### Visceral adiposity-dietary patterns

Two patterns were obtained from RRR, which explained 7.8% of the variations in both the food group intake and the response variables. The first RRR-derived dietary pattern was defined mainly by adjusted-WC (10.8%) and, to a lesser extent, by adjusted-VAI (1.2%). This pattern was characterized by a high intake of soft drinks, organ meat, bread, pasta-rice, and sugars and a low intake of non-leafy vegetables, rye-bulgur, fried vegetables, fruits, leafy vegetables, dried fruits, and non-fermented dairy. The second RRR-derived dietary pattern was explained by more variation in adjusted-VAI (3.3%) but far less variation in adjusted-WC than the first RRR-derived pattern. This pattern had high positive loads on non-leafy vegetables, pickled vegetables, fried vegetables, and bread and high negative loads on eggs, cakes, butter, jam-honey, red meat, poultry, fish, juice, non-fermented dairy, and fruits.

Three DPs were retained by PLS, explaining 17.0% variation in food intake and 7.3% variation in the two response variables. Among the three patterns, most of the adjusted-WC and adjusted-VAI variances were defined by the first and third DPs, respectively. The first pattern represented a higher intake of soft drinks, pasta-rice, bread, and organ meat and a lower intake of non-leafy vegetables, fruits, fried and leafy vegetables, dried fruits, rye-bulgur, legumes, olive oil, fermented dairy, and jam-honey. The second pattern was positively related to the high intake of juice, organ meat, fish, jam-honey, olive oil, canned fruits, nuts, poultry, eggs, dried fruits, non-fermented dairy, fruits, sugars, and red meat and low intake of vegetable fat. The third pattern was a diet with high positive loads on leafy vegetables, non-leafy vegetables, organ meat, soft drinks, olive oil, pickled vegetables, fried vegetables, and bread and high negative loads on biscuits, cakes, butter, eggs, and non-fermented dairy. Factor loading and variances explained by each dietary pattern are presented in Table 1. The median intakes of food groups with factor loading ≥ 0.15 across tertiles of each DP are shown in Supplementary Tables 2, 3.

**TABLE 1:** Factor loadings of food groups in visceral adiposity-related dietary patterns identified by RRR and PLS.

Food groups*^a^*	RRR-derived		PLS-derived		
	Pattern 1	Pattern 2	Pattern 1	Pattern 2	Pattern 3
Soft drinks	**0.42**	0.13	**0.31**	0.09	**0.24**
Organ meat	**0.34**	–0.08	**0.17**	**0.29**	**0.29**
Non-leafy vegetables	**−0.30**	**0.40**	**−0.37**	–0.04	**0.35**
Fried vegetables	**−0.28**	**0.15**	**−0.31**	0.00	**0.18**
Rye-bulgur	**−0.28**	0.13	**−0.25**	–0.01	0.04
Breads	**0.27**	**0.15**	**0.19**	–0.11	**0.15**
Pasta-rice	**0.26**	–0.13	**0.22**	0.02	–0.04
Fruits	**−0.26**	**−0.15**	**−0.33**	**0.19**	0.12
Sugars	**0.21**	–0.12	0.13	**0.19**	0.05
Leafy vegetables	**−0.20**	0.08	**−0.31**	0.08	**0.37**
Dried fruits	**−0.19**	–0.10	**−0.26**	**0.22**	0.10
Non-fermented dairy	**−0.17**	**−0.17**	–0.14	**0.19**	**−0.17**
Biscuits	–0.13	–0.13	–0.04	0.07	**−0.29**
Fast foods	0.11	0.13	0.13	0.02	0.005
Pickled vegetables	–0.11	**0.22**	–0.12	–0.06	**0.20**
Jam-honey	–0.11	**−0.24**	**−0.15**	**0.27**	–0.10
Legumes	–0.10	0.05	**−0.17**	0.04	0.10
Red meat	–0.09	**−0.22**	–0.03	**0.17**	–0.12
Olive oil	–0.07	–0.08	**−0.17**	**0.26**	**0.22**
Fermented dairy	–0.07	–0.04	**−0.15**	0.15	0.14
Juice	0.08	**−0.18**	–0.01	**0.32**	0.12
Canned fruits	0.06	–0.14	0.03	**0.25**	0.05
Fish	0.06	**−0.20**	–0.02	**0.27**	0.10
Animal fat	–0.06	–0.03	–0.07	0.07	0.02
Potatoes	0.05	–0.05	0.004	0.12	0.09
Nuts	–0.05	–0.09	–0.13	**0.24**	0.14
Eggs	–0.05	**−0.37**	–0.02	**0.23**	**−0.19**
Tea-coffee	–0.03	–0.06	–0.03	0.09	0.02
Butter	–0.03	**−0.25**	0.03	0.14	**−0.29**
Cakes	0.02	**−0.28**	0.06	**0.21**	**−0.20**
Poultry	0.02	**−0.20**	–0.02	**0.23**	0.01
Snacks	0.01	0.12	0.13	–0.04	–0.10
Vegetable fat	–0.01	0.14	0.04	**−0.17**	–0.09
Vegetable oil	–0.003	0.04	–0.07	0.08	0.13
**Explained variations (%)**					
Food groups	4.7	3.1	7.3	5.8	3.9
Adjusted_WC*^b^*	10.8	0.36	8.6	1.2	0.9
Adjusted_VAI*^c^*	1.2	3.32	0.69	1.2	2.1
Total responses	6.0	1.84	4.6	1.2	1.5

Factor loadings ≥ 0.15 were bolded for ease of reading. ^*a*^Adjusted for energy intakes using the residual method. ^*b*^Adjusted for age and BMI using the residual method. ^*c*^Adjusted for age using the residual method.

### Association between dietary patterns

**FIGURE 2:**
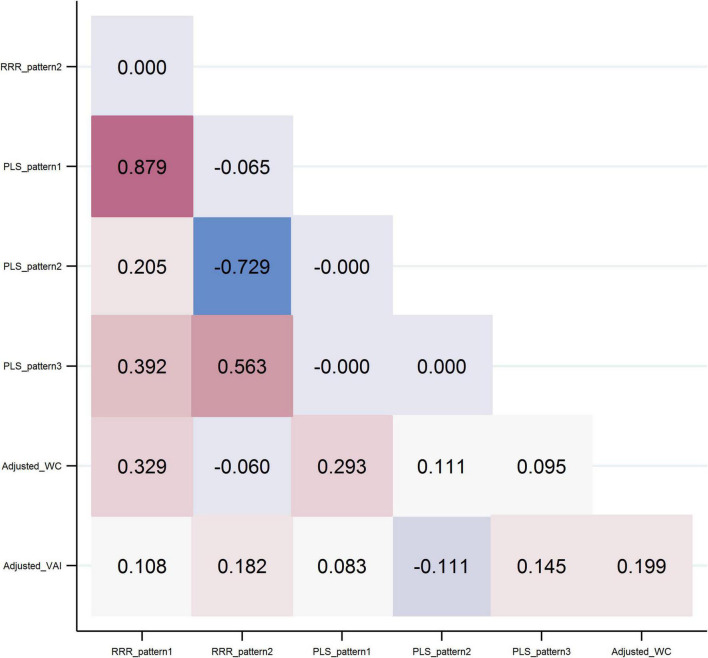
Pearson correlations between dietary patterns and response variables.

In Figure 2, correlations between DPs and response variables are shown. The first pattern derived by each method had a high correlation (*r* = 0.88; *p* < 0.001). RRR-pattern 2 was negatively correlated with the PLS-pattern 2 (*r* = −0.73; *p* < 0.001) and positively correlated with the PLS-pattern 3 (*r* = 0.56; *p* < 0.001). RRR-pattern 2 had a modest inverse correlation with adjusted-WC (*r* = −0.06; *p* = 0.003), and PLS-pattern 2 was inversely correlated with adjusted-VAI (*r* = −0.11; *p* < 0.001). The other patterns were positively correlated with the response variables. It should be mentioned that patterns identified by the same statistical method were uncorrelated.

### Characteristics of participants

The mean age of the 2,496 participants was 38.1 ± 13.3 years at baseline; 1,340 participants were female (53.7%). Characteristics of participants based on tertiles of the first patterns obtained by each method are reported in Table 2. The number of men and smokers increased from tertile 1 to tertile 3 in both patterns. In addition, with increasing tertiles of RRR-pattern 1, participants had higher WC, FPG, and TGs but lower physical activity and HDL-C at baseline. Those with higher adherence to the PLS-pattern 1 were younger and had lower physical activity, BMI, HDL-C, and total cholesterol.

**TABLE 2:** Baseline characteristics of participants according to the tertiles of the first dietary patterns obtained by RRR and PLS.

Variables	Dietary pattern scores tertiles							
	RRR-pattern 1				PLS-pattern 1			
	1st	2nd	3rd	*p*−value*^a^*	1st	2nd	3rd	*p*−value*^a^*
Age, years	38.8 (13.7)	37.8 (13.0)	37.8 (13.2)	0.236	41.0 (13.8)	38.1 (13.1)	35.3 (12.3)	<0.001
Female, *n* (%)	626 (75.3)	477 (57.3)	237 (28.5)	<0.00	605 (72.8)	457 (54.9)	278 (33.4)	<0.001
Academic education, *n* (%)	226 (27.2)	234 (28.1)	198 (23.8)	0.111	220 (26.5)	227 (27.3)	211 (25.3)	0.662
Ever−smokers, *n* (%)	104 (12.5)	167 (20.0)	270 (32.5)	<0.001	109 (13.1)	175 (21.0)	257 (30.9)	<0.001
Premature Family history of CVD, *n* (%)	85 (10.2)	93 (11.2)	75 (9.0)	0.346	83 (10.0)	83 (10.0)	87 (10.4)	0.937
Physical activity, MET-min/wk	1,134 (357, 2,456)	878 (257, 2,292)	834 (119, 2,501)	0.011	1,191 (405, 2,501)	917 (238, 2,233)	792 (99.9, 2,536)	<0.001
BMI, kg/m2	26.8 (4.97)	26.7 (4.91)	26.7 (4.56)	0.873	27.3 (4.86)	26.7 (4.81)	26.3 (4.73)	<0.001
WC, cm	86.5 (13.5)	88.6 (13.3)	91.4 (12.4)	<0.001	88.2 (13.4)	88.5 (13.1)	89.8 (13.1)	0.034
VAI	1.81 (1.12, 2.87)	1.91 (1.27, 3.08)	2.06 (1.27, 3.25)	<0.001	1.93 (1.20, 3.05)	1.91 (1.18, 3.01)	1.94 (1.25, 3.07)	0.806
FPG, mg/dl	86.3 (8.62)	86.9 (8.61)	87.6 (8.60)	0.007	86.9 (9.01)	86.6 (8.40)	87.3 (8.43)	0.256
TGs, mg/dl	108 (77.0, 153)	117 (83.0, 167)	129 (86.0, 182)	<0.001	114 (81.0, 164)	116 (79, 169)	119 (84, 174)	0.129
HDL-C, mg/dl	44.9 (10.6)	42.9 (9.92)	40.5 (9.31)	<0.001	44.4 (10.4)	43.0 (10.1)	40.9 (9.59)	<0.001
Total cholesterol, mg/dl	185 (38.5)	184 (37.8)	185 (37.7)	0.910	188 (38.8)	184 (36.3)	182 (38.7)	0.007
Hypertension^b^, *n* (%)	77 (9.3)	86 (10.3)	93 (11.2)	0.441	94 (11.4)	91 (10.9)	71 (8.6)	0.129
Hypercholesterolemia, *n* (%)	279 (33.6)	273 (32.8)	290 (34.9)	0.663	309 (37.2)	279 (33.5)	254 (30.5)	0.015

Data are shown as mean (SD), median (25th, 75th percentile), or No. (%). ^*a*^Based on ANOVA for normally distributed continuous variables, the Kruskal-Wallis test for non-normally distributed variables, and chi-square for categorical variables. ^*b*^Missing for n = 6. BMI, body mass index; CVD, cardiovascular disease; FPG, fasting plasma glucose; HDL-C, high-density lipoprotein-C; PLS, partial least squares; RRR, reduced rank regression; TGs, triglycerides; VAI, visceral adiposity index; WC, waist circumference.

### Association between dietary patterns and cardiovascular disease events

During a mean of 10.2 (*SD* = 1.94) years of follow-up (25,522 person-years), 141 incidence cases of CVD were identified. HRs (95%CI) for CVD events according to the tertiles of visceral adiposity DPs are shown in Table 3. After adjustment for age, sex, family history of CVD, and energy intake, the risk of CVD was marginally increased per one SD change in the RRR-pattern 2, but the association became non-significant after adjusting for further covariates in model 2. The risk of CVD events was significantly higher in tertiles 2 and 3 of the RRR-pattern 2 than in the first tertile in both adjusted models. One SD increase in the PLS-pattern 3 was associated with a 51% (95%CI = 30–74%) increased risk of CVD events. After controlling for all potential covariates, the estimate was reduced to 19% significantly higher CVD risk per one SD (95%CI = 3–38%; model 2). Consistent with the findings for the continuous score, the risk of CVD events increased by tertiles of PLS-pattern 3 and remained significant after adjusting for all potential covariates (*p*-trend = 0.032). No associations were observed between adherence to the other visceral-adiposity pattern and CVD events.

**TABLE 3:** Hazard ratio (HR) and 95% confidence intervals (CIs) for cardiovascular events after a mean follow-up of 10.2 years based on visceral adiposity-related dietary patterns.

Dietary pattern scores	Continuous		Tertile			
	Per one *SD*	*p*-value	1st	2nd	3rd	*p*-trend
**RRR-dietary patterns**						
Pattern 1						
Incidence cases	141		46	39	56	
Person-years	25,522		8,486	8,610	8,426	
Incidence per 10,000	55.2		54.3	45.3	66.5	
Unadjusted	1.13 (0.97, 1.31)	0.120	1.00	0.83 (0.55, 1.28)	1.22 (0.83, 1.80)	0.312
Model 1	1.07 (0.91, 1.27)	0.375	1.00	0.77 (0.50, 1.19)	1.00 (0.66, 1.52)	0.939
Model 2	1.06 (0.89, 1.25)	0.531	1.00	0.72 (0.47, 1.13)	0.95 (0.62, 1.45)	0.951
Pattern 2						
Incidence cases	141		35	57	49	
Person-years	25,522		8,529	8,456	8,536	
Incidence per 10,000	55.2		41.0	67.4	57.4	
Unadjusted	1.09 (0.91, 1.31)	0.329	1.00	1.64 (1.08, 2.50)	1.40 (0.91, 2.16)	0.148
Model 1	1.20 (1.00, 1.44)	0.054	1.00	1.71 (1.12, 2.61)	1.68 (1.09, 2.60)	0.021
Model 2	1.15 (0.96, 1.38)	0.135	1.00	1.76 (1.15, 2.69)	1.55 (1.00, 2.43)	0.058
**PLS-dietary patterns**						
Pattern 1						
Incidence cases	141		57	38	46	
Person-years	25,522		8,485	8,546	8,490	
Incidence per 10,000	55.2		67.2	44.5	54.2	
Unadjusted	0.95 (0.86, 1.06)	0.339	1.00	0.66 (0.44, 0.99)	0.80 (0.54, 1.18)	0.220
Model 1	1.02 (0.91, 1.14)	0.766	1.00	0.69 (0.45, 1.06)	1.09 (0.72, 1.65)	0.821
Model 2	0.99 (0.88, 1.11)	0.812	1.00	0.65 (0.43, 1.00)	1.03 (0.67, 1.54)	0.959
Pattern 2						
Incidence cases	141		45	42	54	
Person-years	25,522		8,517	8,529	8,476	
Incidence per 10,000	55.2		52.8	49.2	63.7	
Unadjusted	1.04 (0.92, 1.17)	0.512	1.00	0.93 (0.61, 1.42)	1.21 (0.81, 1.79)	0.355
Model 1	0.95 (0.85, 1.07)	0.395	1.00	0.84 (0.55, 1.28)	0.91 (0.61, 1.37)	0.674
Model 2	0.99 (0.88, 1.12)	0.913	1.00	0.88 (0.57, 1.35)	1.01 (0.67, 1.52)	0.970
Pattern 3						
Incidence cases	141		25	43	73	
Person-years	25,522		8,601	8,515	8,405	
Incidence per 10,000	55.2		29.1	50.5	86.8	
Unadjusted	1.51 (1.30, 1.74)	< 0.001	1.00	1.74 (1.06, 2.85)	3.00 (1.90, 4.72)	<0.001
Model 1	1.20 (1.04, 1.40)	0.015	1.00	1.41 (0.86, 2.31)	1.77 (1.07, 1.10)	0.014
Model 2	1.19 (1.03, 1.38)	0.022	1.00	1.34 (0.81, 2.20)	1.64 (1.03, 2.60)	0.032

Model 1: Adjusted for age (continuous), sex, premature family history of CVD (yes/no), and energy intake (continuous). Model 2: Additionally adjusted for academic education (yes/no), ever-smokers (yes/no), physical activity (continuous), baseline prevalent hypertension (yes/no/unclear), baseline prevalent hypercholesterolemia (yes/no), and baseline fasting plasma glucose (continuous).

### Sensitivity analysis

In sensitivity analysis (Table 4), after excluding those with an early occurrence of CVD (<2 years), the risk of CVD events was significantly increased per one SD increase of RRR-pattern 2 and PLS-pattern 3. When we excluded those with early CVD (<2 years) and those younger than 30, the risk of CVD occurrence was 25% (95% CI = 2–53%) and 22% (95% CI = 4–44%) with higher RRR-pattern 2 and PLS-pattern 3, respectively, in the fully adjusted model.

**TABLE 4:** Sensitivity analysis on prospective associations between visceral adiposity-related dietary patterns and risk of cardiovascular events.

	RRR-dietary patterns					PLS-dietary patterns				
	Pattern 1	*p*-value	Pattern 2	*p*-value	Pattern 1	*p*-value	Pattern 2	*p*-value	Pattern 3	*p*-value
**Excluding early CVD (< 2 years)**										
Incidence cases = 119										
Model 1	1.05 (0.88, 1.26)	0.569	1.32 (1.08, 1.61)	0.006	1.02 (0.89, 1.16)	0.808	0.89 (0.79, 1.00)	0.057	1.22 (1.04, 1.43)	0.014
Model 2	1.04 (0.87, 1.24)	0.691	1.25 (1.02, 1.53)	0.029	0.99 (0.87, 1.13)	0.993	0.92 (0.81,1.05)	0.923	1.22 (1.04,1.43)	0.017
**Excluding adults < 30 years**										
Incidence cases = 139										
Model 1	1.08 (0.91, 1.28)	0.377	1.19 (0.99, 1.43)	0.066	1.01 (0.89, 1.14)	0.901	0.96 (0.86, 1.08)	0.517	1.20 (1.04,1.39)	0.015
Model 2	1.06 (0.89, 1.25)	0.515	1.12 (0.93, 1.35)	0.234	0.98 (0.87, 1.10)	0.767	1.00 (0.89, 1.13)	0.942	1.19 (1.03, 1.38)	0.023
**Excluding early CVD and aged < 30 years**										
Incidence cases = 117										
Model 1	1.05 (0.88, 1.27)	0.573	1.31 (1.07, 1.60)	0.008	1.01 (0.88, 1.15)	0.900	0.90 (0.79, 1.02)	0.092	1.23 (1.04, 1.44)	0.013
Model 2	1.04 (0.87, 1.25)	0.673	1.25 (1.02, 1.53)	0.035	0.99 (0.87, 1.13)	0.861	0.93 (0.82, 1.06)	0.286	1.22 (1.04, 1.44)	0.017

Data are shown as hazard ratio (95% confidence intervals) estimated per one SD of dietary pattern scores. Model 1: Adjusted for age (continuous), sex, premature family history of CVD (yes/no), and energy intake (continuous). Model 2: Additionally adjusted for academic education (yes/no), ever-smokers (yes/no), physical activity (continuous), baseline prevalent hypertension (yes/no/unclear), baseline prevalent hypercholesterolemia (yes/no), and baseline fasting plasma glucose (continuous).

## Discussion

In this prospective study, we identified DPs related to visceral adiposity markers using the two methods of RRR and PLS, and then we demonstrated the DPs associations with the risk of CVD in Iranian adults. The first pattern determined by both methods was mainly explained by inter-individual variations in adjusted-WC, explaining 10.8% variation in RRR and 8.6% variation in PLS. These two patterns were positively correlated with the two response variables and were highly correlated with each other (*r* = 0.88). Despite some differences in the magnitude of factor loadings and characteristics of the DPs, both were characterized by higher intakes of soft drinks, bread, pasta-rice, and organ meat and lower intakes of vegetables (fried, leafy, and non-leafy), fruits, dried fruits, and rye-bulgur. After taking into account traditional risk factors for CVD, there was no significant association between these patterns and the development of CVD.

RRR-pattern 2 and PLS-pattern 3 determined more variation in adjusted-VAI than the other patterns. Adjusted VAI was positively correlated with RRR-pattern 2 and PLS-pattern 3, but adjusted-WC showed a small inverse correlation with RRR-pattern 2 (*R* = −0.06) and a positive correlation with PLS-pattern 3 (*r* = 0.10). A moderate positive correlation was found between the two patterns (*r* = 0.56). CVD risk was higher across the tertiles of RRR-pattern 2, but the association of CVD with the score as a continuous variable was not significant. In the fully adjusted model, the risk of CVD was 19% higher per one SD increase in the score of PLS-pattern 3, and the risk of CVD was significantly increased from tertile 1 to tertile 3 of the score. VAI is a lipid combined anthropometric index that takes into consideration BMI, TGs, and HDL-C in addition to WC. Therefore, it is not unexpected that the variable did not exhibit the same correlation with DPs as WC alone.

Limited prospective studies have investigated the association between DPs extracted with RRR and the risk of CVD (10–17); among them, PLS has been used in only two studies (13, 17). The intermediate response variables in these studies were different from ours. Intakes of nutrients were mainly used, and inflammatory markers and some traditional CVD risk factors, including BMI, total cholesterol/HDL-C, and systolic blood pressure, were defined as mediating variables in single studies. In this study, we used surrogates of visceral adiposity as intermediate response variables because of the deleterious effect of visceral adiposity on cardio-metabolic health that has been proposed (19). Since WC has been shown to be ineffective in distinguishing between subcutaneous and visceral fat, we employed two surrogates of visceral adiposity (30). In addition, there is a growing interest in employing the combined indices as compared with the separate anthropometric measures since some research indicates that the combined indices give better insights into the visceral adiposity function than the single variables (31, 32).

The PLS-pattern 3 could explain the 0.9% variation in WC and 2.1% variation in VAI. Therefore, the pattern cannot be considered a major determinant pattern of VAI or WC. However, the effect size of the association was remarkable and increased gradually from tertile 1 to 3 and was significant after controlling for all important traditional risk factors for CVD. The findings imply that even a small variation in visceral adiposity attributed to dietary intake is significantly associated with CVD development. Indeed, in contrast to principal component analysis, both RRR and PLS do not characterize major eating patterns in a population but discover what diet variety is crucial for illness development (33). In this pattern, both healthy (including vegetables and olive oil) and unhealthy foods (including organ meat and soft drinks) were loaded, so the pattern can be considered as a mixed DP. High intake of organ meat, soft drinks, salty vegetables, and fried vegetables may decrease the health benefits of consuming leafy vegetables and olive oil. The study’s findings suggest that the health benefits of consuming vegetables and olive oil depend on the contribution of the other food groups in a dietary pattern. Previous research has also demonstrated the counteractive effects of various foods in a DP, which may modify its overall health benefits or detriments (34).

Taking into account the intermediate variables in the RRR and PLS approaches to studying diet-disease association increases the chance of identifying DPs related to a condition (8, 9). Although the two procedures are conceptually similar, RRR extracts factors explaining the maximal response variation. PLS constructs factors based on maximal variations in both predictors (food groups) and responses (9, 35). It is not well defined which method has the most capability to identify DPs associated with a disease. As expected, in our study, the food group variations were explained more in PLS than in RRR, but the differences in defining the response variables were small. In addition, the number of patterns related to CVD was similar, but the association was more robust and significant for one SD increase in pattern scores derived by PLS. Most previous studies suggest that RRR produces more patterns related to an outcome than PLS (13, 33, 36, 37). However, in a case-control study, PLS could identify more patterns that were significantly associated with CVD than RRR (35). A recent prospective study suggested that deriving a DP for RRR and PLS is similarly efficient (17).

The study’s strengths include a prospective design, a relatively long period of follow-up, administration of a valid FFQ, use of trained interviewers to fill the FFQ, accurate detection of CVD mortality and morbidity with medical document evaluation by a trained health professional, and adjusting for the most important CVD risk factors. Another advantage of the study is the use of the PLS method in addition to RRR. PLS is rarely implemented in nutritional epidemiology. As a result, the study’s findings give some evidence for comparing the ability of the two distinct hybrid methods of RRR and PLS to detect DPs linked to CVD. Another strength of the research is including two different visceral fat surrogates as response variables. Moreover, to reflect visceral adiposity independent of age and BMI, the residuals of WC regressed on age and BMI, and VAI regressed on age were calculated.

It is also necessary to acknowledge some of the study’s limitations. First, accurate reporting of the food items of FFQ relies on participants’ memory. In addition, participants may add bias by over-reporting eating “good” foods or under-reporting eating “bad” foods. Therefore, the FFQ-assessed food intakes are subject to measurement errors (38). Second, DPs were extracted based on dietary intake data collected at baseline, which could not reflect the long-term dietary intake. Third, visceral fat was not directly measured. Magnetic resonance imaging (MRI) and computed tomography (CT) are the gold standards for measuring visceral adiposity; however, cost and participant burden prevent the use of MRI and CT in population-based studies (30). Fourth, we could not analyze CVD mortality and stroke separately because of the low number of cases and power consideration. Fifth, the division of the original food items into food groups is arbitrary, and changes in food grouping may impact the patterns found (8). We categorized grains into three groups, namely, rye-bulgur, pasta-rice, and bread. Because the fiber of bread typically consumed by Iranians differs less, we combine their intakes into a single category. In addition, we divided dairy intakes into two groups according to the fermentation process and not their fat contents due to the likelihood of underestimation for high-fat dairy intakes. Sixth, the *post hoc* power calculation showed that the power of the study in DPs that we did not find significant associations was insufficient. Therefore, the null hypothesis may be incorrectly accepted, leading to false-negative results.

## Conclusion

In conclusion, using RRR and PLS approaches, we identified two DPs positively associated with CVD development. The pattern obtained by RRR with a high intake of non-leafy vegetables, pickled vegetables, fried vegetables, and bread and a low intake of eggs, cakes, butter, jam-honey, red meat, poultry, fish, juice, non-fermented dairy, and fruits showed an inverse correlation with adjusted-WC and a positive correlation with adjusted-VAI. The risk of CVD was substantially greater in the second and third tertiles of the pattern. The PLS-derived pattern with positive loadings on leafy vegetables, non-leafy vegetables, organ meat, soft drinks, olive oil, pickled vegetables, fried vegetables, and bread and high negative loads on biscuits, cakes, butter, eggs, and non-fermented dairy was positively correlated with both response variables. The risk of CVD increased with each SD increase in the score and throughout its tertiles. Our findings implicate that all DPs with high intakes of vegetables are not necessarily cardio-protective diets. Identification of DPs related to visceral adiposity improves our understanding about the association between diet and risk of CVD, which merits more investigation in future prospective studies.

## Data availability statement

The original contributions presented in the study are included in the article/Supplementary material, further inquiries can be directed to the corresponding author/s.

## Ethics statement

This study was designed according to the Declaration of Helsinki principles; the Ethics Committee of the Research Institute for Endocrine Sciences at the Shahid Beheshti University of Medical Sciences confirmed the study design (IR.SBMU.ENDOCRINE.REC.1400.056). The patients/participants provided their written informed consent to participate in this study.

## Author contributions

NM and FR conceptualized the study. NM analyzed and interpreted the data and wrote the first draft of the manuscript. FR contributed to the literature review and helped in preparing the first draft of the manuscript. MM contributed to the data analysis. PM and FA supervised the analyses and contributed to the critical revision of the manuscript. All authors read and approved the final version of the manuscript.
