# Supplementary material for: Visceral adiposity-related dietary patterns and the risk of cardiovascular disease in Iranian adults: A population-based cohort study
Source: Front Nutr. 2022 Jul 28;9:812701. doi: 10.3389/fnut.2022.812701 (PMC9366611; doi:10.3389/fnut.2022.812701)
Supplement: Supplementary file 1 [file Table_1.DOCX]

**Supplementary Table 1-** Food groupings of food items

| **No.** | **Food Groups** | **Food items** |
| --- | --- | --- |
| 1 | Breads | Different types of traditional bread (including lavash, barbari, sanagak, taftun), baguette, bread toast |
| 2 | Pasta-rice | Pasta, rice, different types of noodles |
| 3 | Potatoes | Potatoes, French-fries |
| 4 | Rye-bulgur | Rye, bulgur |
| 5 | Biscuits | biscuits |
| 6 | Cakes | Yazdi cakes, other kind of cakes and pastries, sweet piroshki |
| 7 | Legumes | Lentils, beans, chickpeas, soybeans, mung beans, lima beans |
| 8 | Red meat | Beef, lamb, ground meat, hamburger |
| 9 | Poultry | Poultry |
| 10 | Fish | Fish, tuna-fish |
| 11 | Fast foods | Sausages, pizza |
| 12 | Organ meat | Organ meats of chicken, lamb and beef |
| 13 | Eggs | Eggs |
| 14 | Fermented Dairy | Yogurt, cheese, dough, kashk |
| 15 | Non-fermented dairy | Milk, chocolate milk, ice cream, cream |
| 16 | Butter | Butter |
| 17 | Olive oil | Olive, olive oil |
| 18 | Animal fat | Animal fat |
| 19 | Vegetable fat | Margarine, hydrogenated vegetable oil |
| 20 | Vegetable oil | Vegetable oil |
| 21 | Fruits | Fresh fruits |
| 22 | Dried fruits | Dried figs, raisin, dried Mulberry, dried Peach, dried apricot, other dried fruits |
| 23 | Juice | Carrot juice, orange juice, apple juice, other fruit juices |
| 24 | Canned fruits | Canned pineapple, other canned fruit |
| 25 | Fried vegetables | Vegetables consumed after frying including mixed leafy vegetables, zucchini, celery, eggplants |
| 26 | Leafy vegetables | Green leafy vegetables, lettuce, spinach, Cabbage, cauliflower |
| 27 | Non-leafy vegetables | tomatoes, cucumber, carrots, squash, pumpkin, green bean, green peas, bell Pepper, spinach, green pepper, onion, garlic, shallot , mushroom |
| 28 | Nuts | Peanuts, almonds, walnuts, pistachios, hazelnuts, roasted seeds |
| 29 | Sugars | Sugar cube, sugar, honey, jam, Gaz, candy, Toffee, Sohan, chocolate, Crème Caramel, Candy, halve, halva-ardeh, noghl |
| 30 | Tea-coffee | Tea, coffee |
| 31 | Snacks | Cheese Puffs, potato Chips |
| 32 | Pickled vegetables | Torshi, Shoor |
| 33 | Jam-honey | Jam, honey |
| 34 | Soft drinks | Soft drink |

| **Food groups (g/d)** | **Dietary pattern score tertiles** | | | **p-value ^b^** |
| --- | --- | --- | --- | --- |
|  | **1^st^** | **2^nd^** | **3^rd^** |  |
| **RRR- pattern 1** | | | | |
| **Direct association** |  |  |  |  |
| Soft drinks | 3.84 (0.28, 9.33) | 9.33 (3.84, 40.0) | 40.0 (9.33, 80.0) | <0.001 |
| Organ meat | 0.51 (0.05, 1.62) | 1.27(0.32, 2.70) | 2.08(1.08, 5.40) | <0.001 |
| Breads | 95.1(55.3, 151) | 115(70.4, 186) | 150(91.8, 243) | <0.001 |
| Pasta-rice | 251(118, 283) | 264(176, 305) | 277(250, 406) | <0.001 |
| Sugars | 12.2(6.10, 24.8) | 16.2(9.08, 30.4) | 19.6(11.1, 36.0) | <0.001 |
| **Indirect association** |  |  |  |  |
| Non-leafy vegetables | 259(163, 343) | 177(113, 271) | 147(90.3, 232) | <0.001 |
| Rye-bulgur | 1.43(0.50, 4.35) | 0.76(0.10, 2.14) | 0.44(0.04, 1.00) | <0.001 |
| Fruits | 371(215, 576) | 271(152, 450) | 201(109, 358) | <0.001 |
| Leafy vegetable | 28.5(14.8, 48.7) | 20.0(10.8, 36.4) | 16.1 (8.38, 30.0) | <0.001 |
| Dried fruits | 1.92(0.66, 5.31) | 1.11(0.40, 3.44) | 0.80(0.27, 2.29) | <0.001 |
| Non-fermented dairy | 156(61.8, 247) | 107(37.3, 233) | 73.2(24.4, 174) | <0.001 |
| **RRR-Pattern 2** | | | | |
| **Direct association** |  |  |  |  |
| Non-leafy vegetables | 137(83.4, 218) | 189(122, 272) | 254(163, 346) | <0.001 |
| Fried vegetables | 21.9(12.7, 37.7) | 25.4(15.4, 44.2) | 28.8(16.5, 49.0) | <0.001 |
| Pickled vegetables | 1.32(0.27, 3.48) | 2.14\|(0.60, 5.82) | 2.97(0.67, 8.57) | <0.001 |
| Breads | 110(61.2, 174) | 128(71.7, 193) | 131(75.6, 220) | <0.001 |
| **Indirect association** |  |  |  |  |
| Eggs | 15.3(7.63, 22.9) | 11.4(7.12, 22.9) | 7.63(2.19, 15.3) | <0.001 |
| Cakes | 12.7(5.72, 26.3) | 9.66(4.55, 18.8) | 6.42(2.89, 13.5) | <0.001 |
| Butter | 3.57(0.83, 10.7) | 2.08(0.21, 7.14) | 0.83(0, 3.57) | <0.001 |
| Jam-honey | 4.01(1.37, 9.71) | 3.15(0.90, 7.71) | 1.67(0.66, 4.86) | <0.001 |
| Red meat | 34.8(9.89, 31.2) | 15.1(8.48, 26.1) | 12.9(6.28, 23.7) | <0.001 |
| Fish | 9.00(4.39, 16.6) | 6.75(3.62, 13.8) | 6.00(3.00, 12.0) | <0.001 |
| Poultry | 24.3(12.1, 40.5) | 24.3(12.1, 36.4) | 16.3(10.0, 30.4) | <0.001 |
| Non-fermented dairy | 127(45.8, 240) | 116(42.5, 235) | 77.8(23.0, 230) | <0.001 |
| Juice | 12.6(3.94, 29.1) | 9.72(2.62, 25.4) | 5.92(0.68, 17.4) | <0.001 |
| Fruits | 303(166, 505) | 283(155, 460) | 254(129, 426) | <0.001 |

**Supplementary Table 2** – Median (25^th^, 75^th^ percentile) of dietary food groups for RRR-derived dietary patterns ^a^

^a^ Food groups with factor loadings ≥0.15. ^b^ Based on the Kruskal-Wallis test.

| **Food groups (g/d)** | **Dietary pattern score tertiles** | | | **p-value ^b^** |
| --- | --- | --- | --- | --- |
|  | **1^st^** | **2^nd^** | **3^rd^** |  |
| **PLS- pattern 1** | | | | |
| **Direct association** |  |  |  |  |
| Soft drinks | 3.84(0, 9.33) | 9.33(6.64, 40.0) | 28.0(9.33, 80.0) | <0.001 |
| Organ meat | 0.72(0.11, 1.84) | 1.31(0.30, 2.79) | 1.69(0.63, 4.41) | <0.001 |
| Breads | 99.4(56.8, 151) | 120(71.0, 184) | 148(85.7, 241) | <0.001 |
| Pasta-rice | 252(122, 280) | 265(179, 312) | 276(250, 413) | <0.001 |
| **Indirect association** |  |  |  |  |
| Non-leafy vegetables | 274(192, 370) | 188(124, 270) | 123(73.7, 193) | <0.001 |
| Fried vegetables | 39.6(23.5, 65.5) | 24.9(15.5, 40.7) | 17.6(8.95, 27.8) | <0.001 |
| Rye-bulgur | 1.62(0.50, 4.67) | 0.63(0.12, 1.92) | 0.40(0, 0.94) | <0.001 |
| Fruits | 407(262, 603) | 274(161, 455) | 169(87.5, 298) | <0.001 |
| Leafy vegetable | 40.6(19.6, 54.9) | 21.2(11.6, 37.0) | 12.2(6.52, 22.4) | <0.001 |
| Dried fruits | 2.64(0.88, 7.17) | 1.17(0.45, 3.29) | 0.61(0.21, 1.69) | <0.001 |
| Jam-honey | 3.86 (1.13, 9.57) | 3.16(0.98, 7.71) | 1.80(0.51, 5.53) | <0.001 |
| Legumes | 12.4(6.28, 24.6) | 9.31(5.09, 18.5) | 7.16(3.71, 13.2) | <0.001 |
| Olive oil | 1.37(0.25, 4.18) | 0.73(0.18, 2.71) | 0.40(0.05, 1.51) | <0.001 |
| Fermented dairy | 320(212, 499) | 281(162, 407) | 230(115, 367) | <0.001 |
| **PLS-pattern 2** | | | | |
| **Direct association** |  |  |  |  |
| Organ meat | 0.56(0.04, 1.57) | 1.34(0.30, 2.50) | 2.24(0.98, 5.60) | <0.001 |
| Fruits | 223(108, 406) | 279(161, 459) | 328(185, 519) | <0.001 |
| Sugars | 12.9(6.16, 25.7) | 15.8(8.97, 31.0) | 19.0(10.9, 34.9) | <0.001 |
| Dried fruits | 0.62(0.18, 1.91) | 1.19(0.46, 3.29) | 2.28(0.82, 5.56) | <0.001 |
| Non-fermented dairy | 69.1(18.9, 198) | 114(40.6, 237) | 139(59.7, 241) | <0.001 |
| Jam_honey | 1.13(0.23, 3.86) | 3.00(0.90, 7.19) | 5.75(2.23, 10.6) | <0.001 |
| Red meat | 12.7(5.96, 23.7) | 14.7(8.71, 25.9) | 18.2(10.5, 32.4) | <0.001 |
| Olive oil | 0.25(0.02, 0.96) | 0.70(0.18, 2.38) | 1.67(0.62, 4.57) | <0.001 |
| Juice | 3.18(0, 10.4) | 8.93(3.15, 20.2) | 17.9(7.45, 46.9) | <0.001 |
| Fish | 4.34(1.98, 9.00) | 6.74(3.75, 13.0) | 11.1(6.37, 19.2) | <0.001 |
| Canned fruits | 1.04(0, 3.13) | 2.09(0.70, 8.46) | 4.75(1.39, 15.3) | <0.001 |
| Nuts | 2.55(0.97, 5.39) | 3.92(2.04, 8.40) | 6.36(3.14, 12.4) | <0.001 |
| Eggs | 7.63(3.56, 15.3) | 7.77(7.12, 19.1) | 15.3(7.63, 22.9) | <0.001 |
| Cakes | 6.10(2.74, 13.4) | 9.71(4.41, 18.9) | 12.5(6.50, 25.0) | <0.001 |
| Poultry | 13.7(8.50, 28.4) | 21.4(12.1, 35.5) | 28.1(14.2, 44.0) | <0.001 |
| **Indirect association** |  |  |  |  |
| Vegetable fat | 22.5(6.13, 23.3) | 11.3(0.75, 22.5) | 6.42(0.49, 22.5) | <0.001 |
| **PLS-pattern 3** |  |  |  |  |
| **Direct association** |  |  |  |  |
| Soft drinks | 9.33 (0.77, 18.7) | 9.33(4.22, 40.0) | 18.7(9.33, 40.0) | <0.001 |
| Organ meat | 0.63(0.12, 1.80) | 1.36(0.28, 2.67) | 1.77(0.72, 5.01) | <0.001 |
| Non-leafy vegetables | 135(82.3, 223) | 188(120, 271) | 250(163, 339) | <0.001 |
| Fried vegetables | 21.5(11.5, 37.7) | 23.7(14.1,42.3) | 31.4(19.0, 51.0) | <0.001 |
| Breads | 109(58.5, 179) | 114(67.9, 185) | 136(81.2, 217) | <0.001 |
| Leafy vegetables | 12.9(6.82, 26.0) | 20.2(11.0, 37.2) | 30.6(18.2, 52.4) | <0.001 |
| Pickled vegetables | 1.23(0.26, 3.57) | 1.76(0.49, 5.33) | 3.51(0.82, 8.79) | <0.001 |
| Olive oil | 0.38(0.05, 1.52) | 0.70(0.16, 2.35) | 1.34(0.33, 4.38) | <0.001 |
| **Indirect association** |  |  |  |  |
| Non-fermented dairy | 133(45.6, 242) | 112(40.8, 233) | 81.4(24.3, 216) | <0.001 |
| Biscuits | 2.86(0.53, 12.0) | 1.14(0.16, 5.43) | 0.52(0.03, 2.53 | <0.001 |
| Eggs | 15.3(7.63, 22.9) | 11.4(7.12, 22.80) | 7.63(3.56, 15.3) | <0.001 |
| Butter | 3.57(0.83, 12.5) | 2.08(0.24, 7.14) | 0.83(0, 3.57) | <0.001 |
| Cakes | 11.9(5.40, 25.2) | 9.21(4.25, 18.9) | 7.27(3.36, 15.0) | <0.001 |

**Supplementary Table 3** – Median (percentiles 25, 75) of dietary food groups for PLS-derived dietary patterns ^a^

^a^ Food groups with factor loadings ≥0.15. ^b^ Based on the Kruskal-Wallis test.
